# Improving Patient Outcomes in Abdominal Surgery

**DOI:** 10.3390/jcm13071993

**Published:** 2024-03-29

**Authors:** Claudia Brusasco, Giada Cucciolini, Andrea Barberis, Carlo Introini, Fabio Campodonico, Francesco Corradi

**Affiliations:** 1 Anaesthesia and Intensive Care Unit, E.O. Ospedali Galliera, 16128 Genoa, Italy; claudia.brusasco@galliera.it; 2 Department of Surgical, Medical, Molecular Pathology and Critical Care Medicine, University of Pisa, 56126 Pisa, Italy; giada.cucciolini@phd.unipi.it (G.C.); francesco.corradi@unipi.it (F.C.); 3 General and Epatobiliar Surgery, E.O. Ospedale Galliera, 16128 Genoa, Italy; andrea.barberis@galliera.it; 4 Urology Unit, E.O. Ospedali Galliera, 16128 Genoa, Italy; carlo.introini@galliera.it

Post-operative acute kidney injury (PO-AKI) is a frequent complication described in 15% of non-cardiac surgeries, 30% of cardiac surgeries, and 52% of patients requiring intensive post-operative care. PO-AKI is associated with electrolyte imbalance, fluid accumulation, and metabolic dysfunctions, leading to a cascade of cardiovascular, respiratory, neurological, infectious, and coagulation disorders [1,2]. The ultimate effects of PO-AKI are an increase in hospital length of stay, occurrence of multiple complications, and long-term morbidity and mortality. In this Special Issue, Claudia Brusasco’s manuscripts describe an innovative vision of the prevention and monitoring of post-operative renal failure, which can occur after major open, laparoscopic, and robotic abdominal surgery.

If we apply the most recent Kidney Disease Improving Global Outcomes (KIDGO) criteria [3], the incidence of PO-AKI in non-cardiac surgery is much higher than previously reported, i.e., 64 vs. 15% [4,5,6]. One possible reason for this difference is that mild-to-moderate acute kidney injury (KIDGO stage 1) may often go unrecognized, as it is generally transitory and spontaneously resolves in approximately two-thirds of cases within 7 days of surgery. Nevertheless, this condition should be recognized and managed early, because repeated events of mild PO-AKI can lead to a permanent deterioration in renal function and one-third of KIDGO stage 1 cases—those in which renal function is not restored within seven days of surgery—present a 10% increase in 1-year mortality risk [7], comparable to the 1-year mortality risk in patients who develop more severe PO-AKI (KIDGO stage 2 or 3) resolving within the first seven post-operative days. Finally, the 1-year mortality risk increases by 35% in patients discharged with even a mild-to-moderate degree of persistent acute kidney injury (lasting more than 48 h from its onset) or acute kidney disease (acute or subacute damage to and/or loss of kidney function for 7 to 90 days after exposure to an acute kidney injury) [8].

Excluding urinary tract obstructions caused by surgical procedures, the main peri-operative causes of PO-AKI are hypotension and organ hypoperfusion. Hypotensive events with mean arterial pressure below 65 mmHg for more than 20 min or below 50 mmHg for even less time may cause organ hypoperfusion [9,10,11]. These events can be managed via fluid bolus to increase stroke volume. Splanchnic hypoperfusion may be a consequence of the pneumoperitoneum pressures required for laparoscopic or robotic surgery [12]. Both excessive fluid infusion and excessive pneumoperitoneum pressure increase the risk of PO-AKI by increasing renal venous congestion and/or reducing renal perfusion, respectively. Both conditions are easily detectable by Doppler ultrasound [13,14]. As glomerular filtration pressure in healthy kidneys is around 10 mmHg, even small increments in intra-abdominal pressure generally used for pneumoperitoneum (10–15 mmHg) can cause a 40–50% decrease in renal perfusion, with both pressure level and the duration of pneumoperitoneum possibly impacting renal function. Although 14 mmHg has been recognized as a threshold above which renal damage may be severe, even lower values can increase the risk of medium-to-long-term complications in patients with apparently preserved renal function but prior episodes of PO-AKI [15,16].

Therefore, maintaining the lowest possible pneumoperitoneum pressure for the whole duration of surgery while keeping an optimal surgical space is crucial for reducing the risk of PO-AKI. This can be achieved by (i) using high-flow insufflators with automatic control of flow to guarantee the stability of intra-abdominal pressure, thus limiting the hemodynamic impact and consequently reducing the need for intra-operative fluid infusions and (ii) maintaining a complete neuromuscular blockage for the entire pneumoperitoneum time. An intra-operative management of laparoscopic interventions for major urologic surgeries with low intra-abdominal pressure (8–10 mmHg) and complete neuromuscular blockage has been shown to be feasible without hindering surgical conditions and to reduce post-operative complications, particularly PO-AKI [17]. Indeed, splanchnic hypoperfusion due to increased intra-abdominal pressure could be minimized or prevented via continuous intra-operative and post-operative amino acid infusion [18,19]. Although administering amino acids allows for increasing renal blood flow and glomerular filtration rates by up to 35%, this can only be achieved in kidneys with sufficient residual recruitable nephron mass. Therefore, in cases of pre-existing structural nephropathy, amino acid infusion may not be able to reduce the incidence of PO-AKI [20]. 

In conclusion, the key elements of correctly managing patients with high risk of PO-AKI are (i) careful pre-operative risk assessment, taking into account co-morbidities that may possibly influence renal function, the glomerular filtration rate, and renal arterial and venous flows assessed by Doppler ultrasound; (ii) an intra-operative management aimed at PO-AKI prevention; and (iii) an early diagnosis with appropriate treatment (Figure 1).

As skillfully explained by Ceresoli et al. [21], inguinal hernia is a widespread pathology, which must lead to a reflection on how to adapt the recommendations of the guidelines to their sustainability. While it is true that the surgical approach is still recommended for asymptomatic and minimally symptomatic hernias, it is also true that the guidelines underline the safety of watchful waiting considering the low risk of complications [22]; the first consideration, therefore, could be to create decision-making flow charts to direct patients into different waiting list priority classes depending on the intensity of the symptoms.

The second aspect concerns the overestimation of hernias that are clinically neither detectable nor (occasionally) symptomatic via ultrasound; in any case, this entails referring the patient for surgery and therefore their inclusion on the surgical waiting list, further increasing waiting times. Indeed, in approximately 40% of cases, ultrasound detects hernias with a negative clinical examination [23]; the question, therefore, is about the correct management of these patients. Correct training is essential for general practitioners and surgeons, who must be aware of these data and of what is reported in the guidelines so as to be able to illustrate, using numerical and objective data, the risk/benefit ratio of both the surgical and conservative treatment to the patient on a case-by-case basis.

Our final reflection is mainly related to the Italian healthcare system, which is entering a period of unprecedented crisis [24]. With growing numbers of patients on surgical waiting lists, possible solutions aimed at reducing waiting times—and, which still needs to be demonstrated, improving post-operative outcomes—could be to create hospitals dedicated solely to abdominal surgery, creating a reverse flow to what is increasingly happening for oncological pathologies, which are directed towards “hub” centers. A potential alternative, which may be a more complex choice in a primarily public healthcare system like the Italian one, would be to find a way to deviate asymptomatic patients who still intend to undergo surgery towards the private system.

Considering the magnitude and the implications of abdominal surgery not only for individual health but also for society as a whole and for clinical work, this remains a topic of great interest and relevance which should be taken into consideration when issuing specific clinical recommendations.

## Figures and Tables

**Figure 1:**
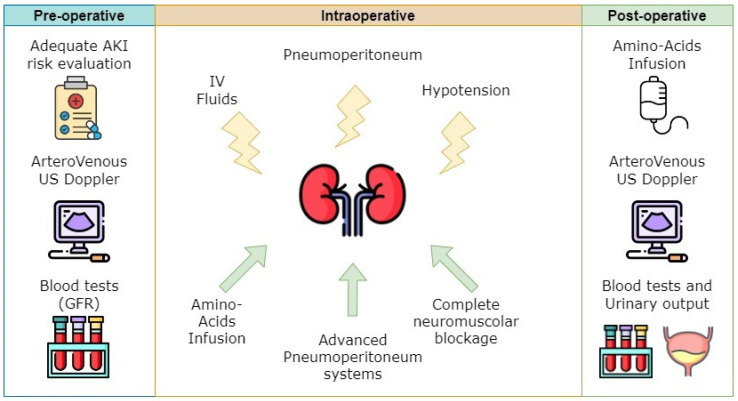
Peri-operative management of post-operative acute kidney injury patients. Yellow lightning bolts are factors impairing kidney perfusion and green arrows are protecting factors.
